# Delta neutrophil index as a promising prognostic marker of emergent surgical intervention for acute diverticulitis in the emergency department

**DOI:** 10.1371/journal.pone.0187629

**Published:** 2017-11-01

**Authors:** Hee Seung Kang, Yong Sung Cha, Kyung Hye Park, Sung Oh Hwang

**Affiliations:** 1 Department of Emergency Medicine, Yonsei University Wonju College of Medicine, Wonju, Republic of Korea; 2 Department of Medical Education, Yonsei University Wonju College of Medicine, Wonju, Republic of Korea; University Hospital Llandough, UNITED KINGDOM

## Abstract

**Background:**

Early identification of patients with acute diverticulitis who require emergent surgical intervention in the emergency department (ED) is important to the physician. Although computed tomography (CT) has an important role in evaluating the severity of diverticulitis, its findings alone may not predict the need for emergent surgical intervention in all patients with acute diverticulitis in the ED. Serum inflammation markers may help to differentiate severity of acute diverticulitis and predict the need for surgical intervention in clinical practice. No information is currently available on the clinical usefulness of the delta neutrophil index (DNI), with respect to the prediction of emergent surgical intervention in patients with acute diverticulitis at the ED.

**Methods:**

This was a retrospective observational study of consecutive adult patients with acute diverticulitis confirmed by CT in the ED, between January 2014 and December 2016. Recruited patients were divided into two groups: emergent surgical intervention and no surgical intervention. The following laboratory serum parameters were examined in the ED: DNI value, C-reactive protein (CRP) levels, white blood cell count, neutrophil count, and neutrophil-to-lymphocyte ratio (NLR). The patients were also examined for the presence or absence of complications by CT.

**Results:**

A total of 132 patients were finally included in the study, with the emergent surgical intervention group constituting 52 patients. The median DNI value, CRP levels, neutrophil count, and NLR were significantly higher in the emergent surgical intervention group than in the no surgical intervention group. The area under the curve for predicting emergent surgical intervention, using the DNI was significantly higher than that of CRP levels, neutrophil count, or NLR. Moreover, the combination of initial DNI and CT was most powerful diagnostic modality.

**Conclusions:**

DNI values measured at the ED combined with CT were good predictors for emergent surgical intervention in acute diverticulitis. If the DNI value is greater than 0.7% and complications in CT are suspected in patients suspected to have acute diverticulitis, the need for emergent surgical intervention should be considered carefully in the ED.

## Introduction

Diverticulosis is a common condition affecting one-third of adults over the age of 45 and above to two-thirds of those over the age of 85 [1]. Diverticulitis is defined as the inflammation or infection of the diverticula-containing colonic sac [2]. Acute diverticulitis develops in 10–25% of adults, with diverticulosis occurring during their lifetime [3]. Recently, the number of younger patients admitted with acute diverticulitis have been increasing [4]. Approximately 10–15% of all patients with acute diverticulitis also present with complications such as abscess, fistulae, and perforation [5]. Although conservative treatment including antibiotics can be successful in most cases of uncomplicated diverticulitis, complicated diverticulitis may require emergency or urgent surgical intervention. The incidence of complicated diverticulitis is reported to be rapidly increasing since the 1990s in the United States [6,7].

In the emergency department (ED), early identification of patients with acute diverticulitis who require emergent surgical intervention can be essential for decision-making [8]. Computed tomography (CT) plays a very important role in confirming diverticulitis, evaluating complications, and excluding alternative diagnoses in the ED [9,10]. However, findings of CT alone may not predict the need for emergent surgical intervention in all patients with acute diverticulitis in the ED. Serum inflammation markers, such as C-reactive protein (CRP) levels, white blood cell (WBC) count, and neutrophil-to-lymphocyte ratios (NLR), may help to differentiate severity of acute diverticulitis and predict the need for surgical intervention in clinical practice [9,11–13].

A new marker, the delta neutrophil index (DNI), has been studied recently. The number of immature granulocytes increase in infection. However, it difficult to measure these numbers in clinical practice because manual counting is inaccurate [14–17]. Nahm et al. showed that the DNI, which is the difference in leukocyte subfractions identified using a cytochemical myeloperoxidase reaction and a nuclear lobularity assay, and is determined using a blood cell analyzer (ADVIA 120, Siemens, Inc.), is strongly correlated with manual immature granulocyte counts [18–20]. We hypothesized that the DNI might be elevated when there is a need for emergent surgical intervention in patients with acute diverticulitis. However, no information is currently available on the clinical usefulness of DNI for predicting a need for emergent surgical intervention in patients presenting to the ED with acute diverticulitis.

Therefore, the primary aim of this study was to assess the accuracy of DNI values at initial presentation in the ED, in predicting the need for emergent surgical intervention, in patients with acute diverticulitis.

## Materials and methods

### Study setting and population

This was a retrospective observational study of consecutive patients older than 18 years with acute diverticulitis confirmed by CT in the ED between January 2014 and December 2016. The ED is located in a single suburban, tertiary-care hospital (Wonju, Republic of Korea), has more than 44,000 annual visits, and is staffed 24 hours a day by board-certified emergency physicians. Due to the retrospective and observational nature of the study, we did not require the informed consent of the participants. Approval for this study was obtained from the Institutional Review Board of Yonsei University Wonju College of Medicine (approval number: CR317030).

The computerized hospital records of patients were searched for those that had ED discharge code words related to all types of colonic diverticulosis or diverticulitis alone and these were initially considered for patient selection. A confirmed diagnosis of acute diverticulitis was made according to CT findings. The CT scan had to have been performed on the day of presentation at the ED. CT diagnostic criteria for acute diverticulitis were the presence of diverticula in the colon, localized colonic wall thickening, surrounding fat stranding, free fluid, abscess formation, or extraluminal air [21].

The exclusion criteria for the study were: 1) age less than 19 years; 2) diverticulitis not confirmed by CT (clinical diagnosis); 3) presence of hematologic abnormalities or other concurrent infections, or administration of granulocyte colony-stimulating factors, glucocorticoids, or other immunosuppressants before study enrollment, any of which could cause changes in the DNI value; 4) transfer from other hospitals due to the effect of other treatments, including antibiotics; 5) transfer to another hospital after ED admission because we could not know the clinical course of the patient; 6) discharged against medical advice; and 7) although a surgical intervention is needed, the treatment is refused.

The included patients were divided two groups: emergent surgical intervention or no surgical intervention. The definition of emergent surgical intervention for acute diverticulitis was the need to perform surgery within 24 hours of ED arrival; indication for surgical intervention consists of signs of abscess, fistula, perforation, generalized peritonitis, and unresponsiveness to initial conservative treatment [22].

### Data collection

Data were collected through retrospective reviews of electronic medical records of patients by one emergency physician who was blinded to the study objectives and hypothesis. The evaluator was also blinded to the categorization of the patient groups and was trained prior to data collection to reduce possible bias from the data collection procedure. We used specific case report forms in this study. The chart evaluator and study coordinator met periodically to resolve any disputes and to review coding rules. The study coordinator monitored the performance of the evaluator. This study was performed retrospectively and observationally, and patient records and information were anonymized prior to analysis.

The following information was obtained from medical records: age; sex; presence of diabetes mellitus; initial vital signs (systolic blood pressure, heart rate, respiratory rate, and body temperature); initial symptoms including nausea/vomiting, fever/chills, diarrhea, or abdominal pain; abdominal physical examination findings (abdominal tenderness or rebound tenderness); and treatment including emergent surgical intervention or no surgical intervention. The included patients were examined for the presence or absence of complications by CT findings based on the modified Hinchey classification [23]. Patients with class Ia (confined pericolic inflammation) were classified as uncomplicated patients with no evidence of an abscess, perforation, or fistula on CT images. Patients who presented with class Ib (confined pericolic abscess), II (pelvic, distant intra-abdominal, or retroperitoneal abscess), III (generalized purulent peritonitis), or IV (generalized fecal peritonitis) diverticulitis were classified as complicated patients. The distinction between class III and IV was based on the findings during laparotomy, if surgery was performed for generalized peritonitis after diagnosis using CT. The following laboratory serum parameters were checked in the ED: DNI values, CRP levels, WBC counts, neutrophil counts, and NLR.

The ADVIA 120/2120 automatic cell analyzer (Siemens, Tarrytown, NY, USA) was used for determining DNI values. It is a specific technology found in the ADVIA unit manufactured by Siemens, which is a flow cytometry-based hematologic analyzer that uses two independent WBC analysis methods using a myeloperoxidase (MPO) channel and a lobularity/nuclear density channel. DNI values were calculated in leukocyte differentials using the following formula: DNI = (the leukocyte subfraction assayed in the MPO channel by cytochemical reaction)–(the leukocyte subfraction counted in the nuclear lobularity channel by the reflected light beam) [18,24].

### Study endpoints

Primary endpoints in this study were to evaluate differences in DNI values between emergent surgical intervention group and no surgical intervention group and whether DNI could be a useful addition to improve the accuracy of CT in predicting the need for emergent surgical intervention, in patients with acute diverticulitis at the ED.

### Data analysis

Categorical variables were presented as frequencies and percentages, and continuous variables as means and standard deviations, or as medians and interquartile ranges. Normality was assessed using the Shapiro–Wilk test. The chi-square test or Fisher’s exact test was used to compare categorical variables, while either the two-sample t-test or the Mann–Whitney U test were used to compare continuous variables. Variables with p values of < 0.05 by univariate analysis were entered into the multiple logistic regression analysis to identify early predictors for emergent surgical intervention. The area under the curve (AUC) for the predictive ability of emergent surgical intervention was determined using receiver operating characteristic (ROC) curves. In addition, comparisons of AUCs were used to compare the predictive ability of each method for emergent surgical intervention. Optimal cut-off points of predictors were evaluated using ROC curves and the youden index. P values of < 0.05 were considered statistically significant, and the analysis was performed using SPSS Ver. 23 (IBM, Armonk, NY, USA) and MedCalc Statistical Software version 17.5.3 (MedCalc Software, Ostend, Belgium).

## Results

### Characteristics of study subjects

A total of 162 consecutive acute diverticulitis patients were treated during the study period. Some patients were excluded for the following reasons: 7 patients did not undergo CT (clinical diagnosis); 2 patients had a hematologic malignancy; 10 patients were transferred from other hospitals after using antibiotics; 4 patients were transferred to another hospital; although a surgical intervention was needed, it was refused in 2 patients; and insufficient data was available for 5 patients. Therefore, 132 patients were finally included.

The baseline characteristics of the 132 study participants are shown in Table 1. Men constituted 54.5% of the study group, and the overall median age was 56 years. The most common symptom presented at the ED was abdominal pain. Abdominal tenderness and rebound tenderness was observed in 88.6% and 43.2% of patients, respectively. The ascending colon (67.4%) was the most common site for acute diverticulitis. The severity of patients classified by the modified Hinchey classification were Ia (57.6%), Ib (6.8%), II (5.3%), III (13.6%), and IV (16.7%), respectively.

**Table 1:** Baseline characteristics of patients with acute diverticulitis.

Variables	Total (n = 132)	No surgical intervention (n = 80, 60.6%)	Emergent surgical intervention (n = 52, 39.4%)	p-value
Age	56 (41–71)*	48 (35–57)*	69 (55–76)*	<0.001
Male sex	72 (54.5%)	41 (51.3%)	31 (59.6%)	0.346
Diabetes mellitus	17 (12.9%)	7 (8.8%)	10 (19.2%)	0.079
Vital signs				
SBP (mmHg)	140 (121–153)*	141 (125–155)*	130 (114–149)*	0.078
HR (beats/minute)	94 (83–106)*	90 (83–103)*	100 (85–113)*	0.011
RR (beats/minute)	20 (18–20)*	19 (18–20)*	20 (18–20)*	0.128
Body temperature (°C)	36.9 (36.5–37.5)*	36.8 (36.5–37.4)*	36.9 (36.5–38)*	0.195
Symptoms				
Nausea/vomiting	24 (18.2%)	17 (21.3%)	7 (13.5%)	0.257
Fever/chills	45 (34.1%)	22 (27.5%)	23 (44.2%)	0.048
Diarrhea	30 (22.7%)	17 (21.3%)	13 (25%)	0.615
Abdominal pain	123 (93.2%)	73 (91.3%)	50 (96.2%)	0.482
Physical examinations				
Abdominal tenderness	117 (88.6%)	70 (87.5%)	47 (90.4%)	0.610
Abdominal rebound tenderness	57 (43.2%)	28 (35%)	29 (55.8%)	0.019
Location of diverticulitis				< 0.001
Ascending colon	89 (67.4%)	67 (83.8%)	22 (42.3%)	
Descending colon	6 (4.5%)	1 (1.3%)	5 (9.6%)	
Sigmoid colon	37 (28%)	12 (15%)	25 (48.1%)	
Presence of complication identified by CT	56 (42.4%)	6 (7.5%)	50 (96.2%)	< 0.001
Modified Hinchey classification				< 0.001
Ia	76 (57.6%)	74 (92.4%)	2 (3.8%)	
Ib	9 (6.8%)	3 (3.8%)	6 (11.5%)	
II	7 (5.3%)	3 (3.8%)	4 (7.7%)	
III	18 (13.6%)	0 (0%)	18 (34.6%)	
IV	22 (16.7%)	0 (0%)	22 (42.3%)	
Delta neutrophil index (%)	0.6 (0–2.4)	0 (0–0.3)	2.6 (1.93–5.58)	<0.001
C-reactive protein levels (mg/dL)	6.32 (1.93–13.7)*	4.28 (1.29–9.4)*	11.25 (3.21–18.8)*	0.001
White blood cell count (per μL)	11346 ± 4738†	11165 ± 3629†	11623 ± 6094†	0.627
Neutrophil count (per μL)	9139 ± 4383†	8401 ± 3497†	10274 ± 5315†	0.028
Neutrophil-to-lymphocyte ratio	5.48 (3.66–10.13)*	4.83 (3.2–6.74)*	10.85 (4.81–15.23)*	<0.001

* median (interquartile range).

† mean ± standard deviation.

SBP: systolic blood pressure, HR: heart rate, RR: respiratory rate, CT: computed tomography.

The emergent surgical intervention group included 52 patients (39.4%). Patients in the no surgical intervention and emergent surgical intervention groups differed significantly in terms of age, heart rate, presence of fever/chills or abdominal rebound tenderness, location of diverticulitis, and presence of complication identified by CT (Table 1).

### Main results

Median DNI values, CRP levels, neutrophil count, and NLRs were significantly higher in the emergent surgical intervention group (0% vs. 2.6%, p < 0.001; 4.28 mg/dL vs. 11.25 mg/dL, p = 0.001; 8401 per μL vs. 10274 per μL, p = 0.028; and 4.83 vs. 10.85, p < 0.001, respectively), but mean serum WBC counts were non-significantly higher in the emergent surgical intervention group (Table 1).

Based on the multiple logistic regression analyses, patient age (odds ratio [OR] 1.037, 95% confidence interval [CI] 1.006–1.069, p = 0.020) and DNI (OR 1.664, 95% CI 1.203–2.301, p = 0.002) (Table 2) were identified as predictive factors to evaluate the need for emergent surgical intervention in patients with acute diverticulitis. Patients with fever/chills were excluded from the multiple logistic regression analyses because patients with fevers/chills may have a higher DNI.

**Table 2:** Predictors of emergent surgical intervention as determined using univariate and multivariate logistic regression analysis.

		Univariate			Multivariate	
Variables	Odds ratio	95% CI	p-value	Odds ratio	95% CI	p-value
Age	1.050	1.027–1.075	< 0.001	1.037	1.006–1.069	0.020
HR	1.028	1.006–1.05	0.013	0.990	0.957–1.024	0.548
RT	2.342	1.146–4.784	0.02	1.549	0.577–4.157	0.385
DNI	2.038	1.505–2.76	< 0.001	1.664	1.203–2.301	0.002
CRP	1.09	1.04–1.141	< 0.001	1.022	0.956–1.092	0.530
Neutrophil	1.000	1.000–1.000	0.018	1.000	1.000–1.000	0.072
NLR	1.194	1.097–1.298	< 0.001	1.019	0.926–1.121	0.704

CI: confidence interval, HR: heart rate, RT: abdominal rebound tenderness, DNI: delta neutrophil index, CRP: C-reactive protein, NLR: neutrophil-to-lymphocyte ratio

**Fig 1:**
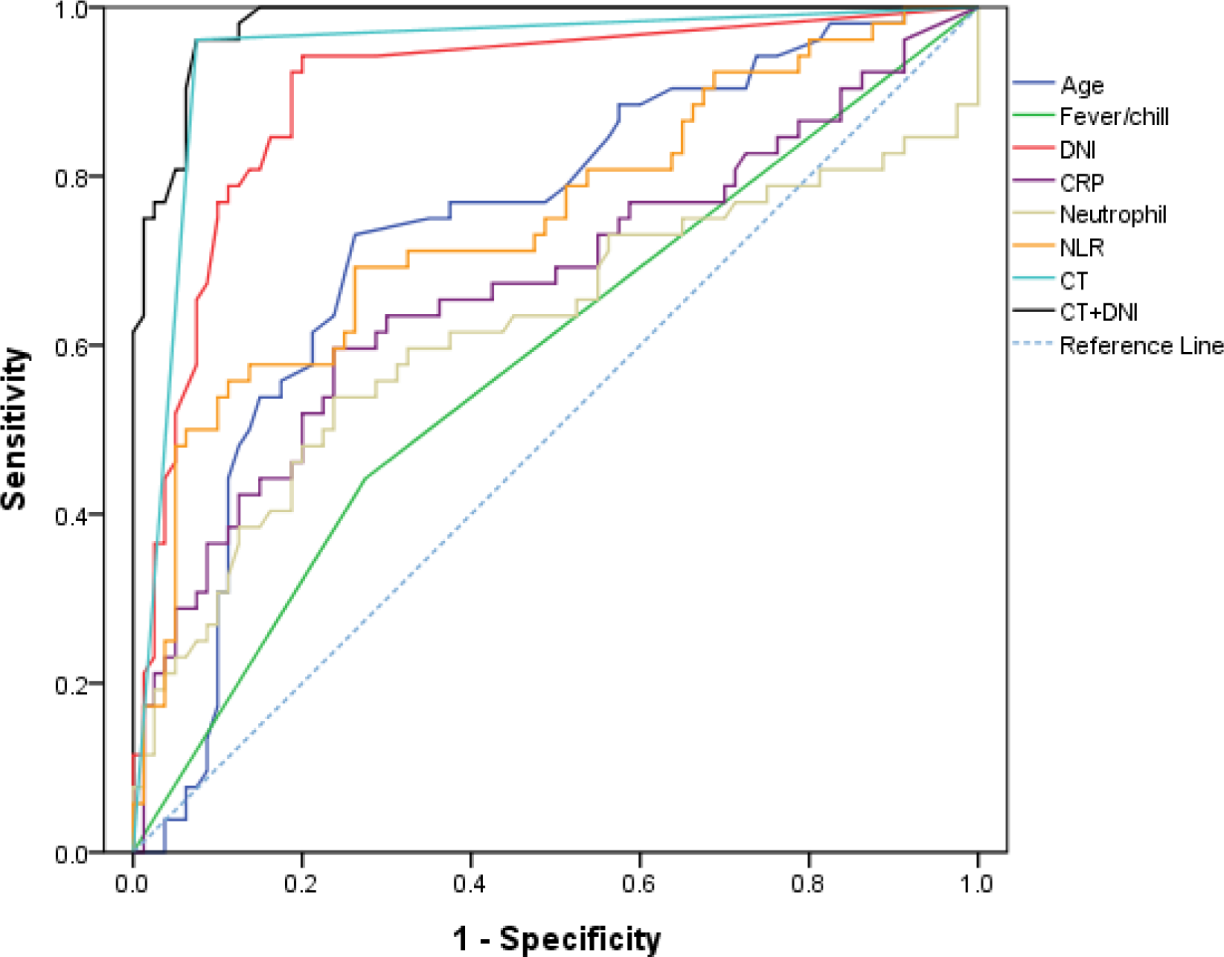
Receiver operating characteristic curves of clinical variables, inflammatory markers, and CT. CT: computed tomography, DNI, delta neutrophil index; CRP, C-reactive protein; NLR, neutrophil-to-lymphocyte ratio.

The AUC for predicting the need for emergent surgical intervention using the DNI was significantly higher than that for age, presence of fever/chills, CRP levels, neutrophil count, or NLRs. The optimum cut-off for DNI values was 0.7% (sensitivity, 94.2%; specificity, 80%) (Table 3, Fig 1). The AUC for predicting emergent surgical intervention using a combination of initial DNI and CT, which is a useful tool for differentiating between simple and complicated diverticulitis, (0.981 [95% CI: 0.941–0.997]) was the most powerful diagnostic modality (Table 3).

**Table 3:** Diagnostic accuracy of clinical variables, inflammatory markers, and CT and comparisons of areas under curves.

Markers	AUC (95% CI)	Cut-off	Sensitivity (95% CI)	Specificity (95% CI)	p-value
DNI	0.902 (0.838–0.947)	> 0.7	94.2 (84.1–98.8)	80 (69.6–88.1)	< 0.001
Age	0.739 (0.655–0.811)	-	73.1 (59–84.4)	73.8 (62.7–83)	0.001
Fever/chill	0.584 (0.495–0.669)	-	44.2 (30.5–58.7)	72.5 (61.4–81.9)	< 0.001
CRP	0.673 (0.586–0.752)	> 9.45	59.6 (45.1–73)	76.3 (65.4–85.1)	< 0.001
Neutrophil	0.619 (0.530–0.702)	10460	53.9 (39.5–67.8)	76.3 (65.4–85.1)	< 0.001
NLR	0.749 (0.666–0.820)	9.158	55.8 (41.3–69.5)	88.8 (79.7–94.7)	< 0.001
CT	0.943 (0.889–0.976)	-	96.2 (86.8–99.5)	92.5 (84.4–97.2)	-
CT+age	0.969 (0.923–0.991)	-	96.2 (86.8–99.5)	93.8 (86–97.9)	0.079
CT+fever/chills	0.943 (0.889–0.976)	-	96.2 (86.8–99.5)	92.5 (84.4–97.2)	0.977
CT+DNI	0.981 (0.941–0.997)	-	96.2 (86.8–99.5)	92.5 (84.4–97.2)	0.007
CT+CRP	0.968 (0.922–0.991)	-	98.1 (89.7–100)	91.3 (82.8–96.4)	0.039
CT+Neutrophil	0.976 (0.933–0.995)	-	100 (93.2–100)	92.5 (84.4–97.2)	0.019
CT+NLR	0.978 (0.936–0.996)	-	98.1 (89.7–100)	92.5 (84.4–97.2)	0.023

CT: computed tomography, AUC: area under curve, CI: confidence interval, DNI: delta neutrophil index, CRP: C-reactive protein, NLR: neutrophil-to-lymphocyte ratio.

## Discussion

In this study, initial DNI upon presentation at the ED was significantly greater in patients with emergent surgical intervention compared to those without emergent surgical intervention. In this study, the predictive power of the DNI with regards to the need for emergent surgical intervention in patients with acute diverticulitis was significantly higher than that of serum CRP levels, and WBC and neutrophil counts, which are commonly used markers for predicting inflammation and infection in the ED. The diagnostic power of the DNI in predicting the need for emergent surgical intervention in patients with acute diverticulitis was also significantly higher than that of the NLR, which was recently reported as being useful for predicting of surgical intervention in acute diverticulitis [13]. Even though CT-based imaging severity criteria, including the Hinchey classification and its successive modifications, have been shown to assist in the prediction of the clinical course of diverticulitis [23,25,26], the predictive value of the combination of initial DNI and CT was significantly higher than its of only CT and the predictive value of combination of initial DNI and CT was most powerful diagnostic modality. Based on this study, patients with a DNI value greater than 0.7% have a greater probability of experiencing emergent surgical intervention due to progressive and complicated diverticulitis. The usefulness of the DNI for predicting the need for emergent surgical intervention in acute diverticulitis can be explained by the idea that the proportion of immature granulocytes in the circulation will increase more when acute diverticulitis progresses to peri-diverticular structures and forms abscesses or perforations, for which surgical intervention is needed. Also, the rationale behind its higher diagnostic power than other serum markers may be that changes in DNI values may precede changes in the absolute number of WBCs or neutrophils because the process of granular leukocyte differentiation in infectious conditions starts from immature granulocyte formation [27]. The DNI can also easily be used to evaluate inflammation and infection in the ED setting, because the tests can be performed at the same time as routine complete blood counts without any additional time or cost. It is acceptable to patients because venipuncture would already routinely be performed. This can be a great advantage in the ED. We suggest that the DNI could be a useful addition to improve the predictive accuracy of CT in acute diverticulitis.

Serum CRP levels are widely used as an objective index of disease activity in ED settings. The concentration of CRP, a plasma protein, rises dramatically as a result of cytokine-mediated responses to most forms of infection, inflammation, and tissue injury [28]. There are reports that patients with acute complicated diverticulitis present with considerably higher CRP levels compared with patients with uncomplicated episodes. However, CRP levels are only helpful as an indicator for the presence of complicated diverticulitis [29,30]. In this study, serum CRP levels were significantly higher in both the emergent surgical intervention group than in the no surgical intervention group. However, the diagnostic power of the DNI was significantly higher than that of serum CRP levels (p < 0.001).

A recent prospective study evaluated the role of the NLR in the setting of acute diverticulitis. They concluded that the NLR was more accurate than CRP levels, and WBC and neutrophil counts in predicting the need for a surgical intervention, which indicates complicated diverticulitis [13]. The principle of the NLR is that neutrophil levels elevate as part of the inflammatory cascade, whereas lymphocytes are decreased during sepsis. Measuring the divergence between these two WBC components has been believed to be more accurate at predicting poor clinical outcomes than measuring each one individually [31]. In this study, the diagnostic power of the NLR for predicting emergent surgical intervention was also higher than that of CRP levels and neutrophil counts. However, the diagnostic power of the NLR was significantly lower than that of the DNI (p < 0.001).

Median age and the presence of fever/chills differed between the patient groups in this study. Patients who required surgical intervention were older, and this may be because older patients are more likely to have atypical symptoms and decreased physiologic defense mechanisms against bowel inflammation and complications. Therefore, older patients may have more complicated diverticulitis and a greater need for emergent surgical intervention. Moreover, as acute diverticulitis progresses, the inflammatory response becomes worse, more likely resulting in fever.

There were some limitations. First, because of the retrospective nature of this study, some types of data may have been missing. Second, since this study was conducted at the emergency center of a single hospital, the sample size was small. In addition, selection bias could have been caused by the exclusion of patients. Nevertheless, we investigated all acute diverticulitis patients who were admitted to this hospital since DNI levels became measurable, in order to reduce possible biases. Third, we did not evaluate the time from symptom onset to ED arrival. It is possible that this time gap could have affected the values of the inflammatory markers. Fourth, because serial DNI values were not investigated after admission, we did not evaluate their usefulness for evaluating treatment responses by assessing changes in inflammatory markers after definitive treatment. Fifth, under certain conditions, patients with acute diverticulitis, such as those with diffuse peritonitis, should immediately be sent to an operating room. The use of DNI may not influence clinical decision making in these cases. A prospective study is needed to exclusively investigate the predictive value of DNI in patients not requiring surgery.

## Conclusions

DNI values measured at the ED combined with CT were good predictors of the need for emergent surgical intervention in acute diverticulitis. If the DNI value is greater than 0.7% and complications in CT are suspected in patients suspected to have acute diverticulitis, the need for emergent surgical intervention should be considered carefully in the ED.
